# Osteoarticular Expression of Musashi-1 in an Experimental Model of Arthritis

**DOI:** 10.1155/2015/681456

**Published:** 2015-05-03

**Authors:** Francisco O'Valle, Magdalena Peregrina, Vicente Crespo-Lora, Pablo Galindo-Moreno, Maria Roman, Miguel Padial-Molina, Francisco Mesa, Jose Aneiros-Fernandez, David Aguilar, Elena Gonzalez-Rey, Mario Delgado, Pedro Hernandez-Cortes

**Affiliations:** ^1^Department of Pathology and IBIMER, School of Medicine, University of Granada, 18012 Granada, Spain; ^2^The Spanish Institute of Social Security (INSS), 18006 Granada, Spain; ^3^Oral Surgery and Implant Dentistry Department, School of Dentistry, University of Granada, 18017 Granada, Spain; ^4^Plastic Surgery Department, Virgen de las Nieves University Hospital, 18014 Granada, Spain; ^5^Periodontics Department, School of Dentistry, University of Granada, 18017 Granada, Spain; ^6^Parasitology and Biomedicine López-Neyra Institute, CSIC, 18016 Armilla, Granada, Spain; ^7^Orthopedic Surgery Department, San Cecilio University Hospital of Granada, 18012 Granada, Spain

## Abstract

*Background*. Collagen-induced arthritis (CIA), a murine experimental disease model induced by immunization with type II collagen (CII), is used to evaluate novel therapeutic strategies for rheumatoid arthritis. Adult stem cell marker Musashi-1 (Msi1) plays an important role in regulating the maintenance and differentiation of stem/precursor cells. The objectives of this investigation were to perform a morphological study of the experimental CIA model, evaluate the effect of TNF*α*-blocker (etanercept) treatment, and determine the immunohistochemical expression of Msi1 protein. *Methods*. CIA was induced in 50 male DBA1/J mice for analyses of tissue and serum cytokine; clinical and morphological lesions in limbs; and immunohistochemical expression of Msi1. *Results*. Clinically, TNF*α*-blocker treatment attenuated CIA on day 32 after immunization (*P* < 0.001). Msi1 protein expression was significantly higher in joints damaged by CIA than in those with no lesions (*P* < 0.0001) and was related to the severity of the lesions (Spearman's rho = 0.775, *P* = 0.0001). *Conclusions*. Treatment with etanercept attenuates osteoarticular lesions in the murine CIA model. Osteoarticular expression of Msi1 protein is increased in joints with CIA-induced lesion and absent in nonlesioned joints, suggesting that this protein is expressed when the lesion is produced in order to favor tissue repair.

## 1. Introduction

Collagen-induced arthritis (CIA), a murine experimental disease model induced by immunization with type II collagen (CII), shares a number of clinical, histopathological, and immunological features with rheumatoid arthritis (RA) [1]. Although its etiology is unknown, the initial stages of RA and CIA involve multiple steps that can be divided into two main phases: the initiation and establishment of autoimmunity to collagen-rich joint components, and later events associated with progressively destructive inflammatory processes [1–4].

Progression of the autoimmune response involves the development of autoreactive Th1 and Th17 cells, their entry into the joint tissues, and the subsequent recruitment of inflammatory cells via multiple mediators [4]. The chronic nature of the inflammatory process in RA suggests a disturbance of immune regulation in the joint, probably caused by an excessive inflammatory response along with deficiency in the mechanisms controlling the immune response. Available therapies are based on immunosuppressive agents that inhibit the inflammatory component of RA and either reduce the relapse rate or delay disease onset. However, they have multiple effects, some of which are undesirable, and in the long term they do not prevent progressive clinical disability [5].

Tumor necrosis factor alpha (TNF-*α*) is a proinflammatory cytokine expressed in the pannus of the inflamed joint in RA [6–9]. TNF*α*-blocker drugs are among those currently used for the treatment of RA. The subcutaneous administration to RA patients of the TNF-*α* receptor antagonist etanercept (50 mg once a week) was found to induce clinical improvements not observed with the drugs previously used in this disease [10, 11]. In addition, the beneficial effects of TNF*α*-blocker therapy have been demonstrated by various research groups in a mouse model of CIA [12–17].

The adult stem cell marker Musashi-1 (Msi1) is an RNA-binding protein of 362 amino acids with two ribonucleoprotein motifs (RBD1 and RBD2) [18] of 39 kDa molecular weight. Msi1 is associated with the maintenance and asymmetric cell division of neural and epithelial progenitor cells [19]. It is expressed in various epithelial stem cells and plays an important role in regulating the maintenance and differentiation of stem/precursor cells [20, 21]. Msi1 is known to regulate progenitor cell function through the posttranscriptional regulation of its target RNA [20]. Msi1 also acts as an important positive regulator of cell proliferation and inhibitor of apoptosis by reducing Notch-1 expression [22].

Modulation of Msi1 immunohistochemical expression has been identified in a murine model of inflammatory colitis [23], and it has been speculated that Msi1 might promote cell proliferation by accelerating the cell cycle in neoplastic cells [24], suggesting a role for this protein in tissue repair in different processes and as a potential therapeutic target in regenerative medicine.

The objectives of this study were to morphologically analyze the experimental collagen-induced arthritis model, to evaluate the effect of treatment with a TNF*α*-blocker (etanercept), and to determine the immunohistochemical expression of Msi1 protein.

## 2. Material and Methods

### 2.1. Induction and Treatment of Collagen-Induced Arthritis (CIA)

CIA was induced in 50 male DBA1/J mice (7 to 10 weeks old; Jackson Laboratories, Bar Harbor, ME) by subcutaneous injection into the tail base with 200 *μ*g bovine CII (Sigma, St. Louis, MO) at day 0 and with 100 *μ*g CII at day 21, both emulsified in complete Freund's adjuvant containing 200 *μ*g* Mycobacterium tuberculosis* H37 RA (Difco, Detroit, Michigan). Treatment consisted of the intraperitoneal injection of 2 mg of etanercept (Enbrel, Wyeth Europa Ltd., UK; E-group) or of phosphate-buffered saline (PBS; untreated control, C-group) once a week for four weeks (on days 25, 32, 39, and 46) starting at day 25 after immunization, when all mice showed established arthritis (clinical score >2). Mice were evaluated by two independent blinded examiners every other day and monitored for signs of arthritis onset according to the following clinical score: grade 0, no swelling; grade 1, slight swelling and erythema; grade 2, moderate swelling and edema; grade 3, extreme swelling and pronounced edema; or grade 4, joint rigidity. Each limb was graded, giving a maximum possible score of 16 per animal. Paw swelling was assessed by measuring the thickness of the affected hind paws with 0 to 10 mm calipers (on days 25, 32, 39, 46, and 53). All experiments were performed in a European Union-certified laboratory following national guidelines for the ethical care of animals (RD 53/2013, EU Directive 63/2010).

### 2.2. Cytokine Determination

Protein extracts were isolated by homogenization of joints (50 mg tissue/mL) in 50 mM Tris-HCl, pH 7.4, with 0.5 mM DTT and proteinase inhibitor cocktail (10 *μ*g/mL, Sigma) for cytokine determination in joints, and serum samples were collected at the disease peak (day 40); serum and joint cytokine and chemokine levels were determined by specific sandwich ELISAs using capture/biotinylated detection Abs from BD Pharmingen (San Diego, CA) according to the manufacturer's recommendations.

### 2.3. Histopathological Study

For the histopathological study, mice were anesthetized with ether and sacrificed by cervical dislocation at day 15 (*n* = 20), 21 (*n* = 20), or 28 (*n* = 10) after commencement of treatment with etanercept or PBS (i.e., days 39, 46, and 53 after immunization). They were fixed in 10% buffered formalin for 24 hours, decalcified with Decalcifier I, containing formaldehyde (10% w/v), formic acid (8% w/v), and methanol (1% w/v) (Surgipath softener I Europe Ltd., Peterborough, UK) for 24 h in oven at 37°C. Next, the four limbs were sectioned longitudinally, dehydrated with alcohol, and embedded in paraffin in an automatic tissue processor Excelsior ES (Thermo Scientific, CA, USA); 4 *μ*m sections were stained with hematoxylin and eosin (H&E) or Masson trichrome stain. Histopathological changes were scored in a blinded manner based on cell infiltration, cartilage destruction, and bone erosion parameters as previously described [25]. Cell infiltration was scored on a scale of 0–3 according to the number of affected joints (0: none, 1: <2, 2: 3–5; 3: >5 joints), and the amount of inflammatory cells in the synovial cavity (exudate) and synovial tissue (infiltrate) was also recorded. Cartilage destruction was graded on a scale of 0–3, ranging from the appearance of dead chondrocyte (empty lacunae) to complete loss of articular cartilage. Bone erosions were graded on a scale of 0–3, ranging from normal bone appearance to fully eroded cortical bone structure in patella and femur condyle. Pannus and involvement of the bone marrow and/or soft tissue were treated as dichotomous variables (present or absent).

### 2.4. Immunohistochemical Analysis

Decalcified and paraffin-embedded sections were dewaxed, hydrated, and heat-treated in 1 mM EDTA pH 8 in an antigen retrieval PT module (Thermo Fisher Scientific Inc., Waltham, MA) at 95°C for 20 min. Sections were incubated for 16 h at 4°C with the prediluted polyclonal antibody against Musashi-1 (Sigma-Aldrich, Barcelona, Spain) at 1 : 100 dilution to identify cellular expression. An automatic immunostainer (Autostainer 480, Thermo Fisher Scientific Inc.) was used for the immunohistochemical study, applying the peroxidase conjugated micropolymer method and developing with diaminobenzidine (Ultravision Quanto, Master Diagnóstica, Granada, Spain). Expression was assessed semiquantitatively on a scale of 0 to 3 (0: absence, 1: mild [<10% positive cells], 2: moderate [10 to 25%], 3: intense [>25%] in bone tissue, hyaline cartilage, joint capsule, synovium, ligaments, striated muscle cells, endothelial cells, and adipocytes). The variables were subsequently categorized in two groups (presence/absence of osteoarticular lesion), calculating the total Msi1 expression score for each group.

### 2.5. Statistical Analysis

SPSS 20.0 (IBM Inc., Chicago, IL) was used for the statistical analysis. The normality of the distribution of variables was examined with the one-dimensional Kolmogorov-Smirnov test. Results were expressed as mean ± standard deviation for normally distributed continuous variables and frequencies for categorical variables. The bivariate tests and Spearman correlation coefficient used are reported in the table footnotes. A *P* value of 0.05 was accepted as the statistical significance threshold.

## 3. Results

**Figure 1:**
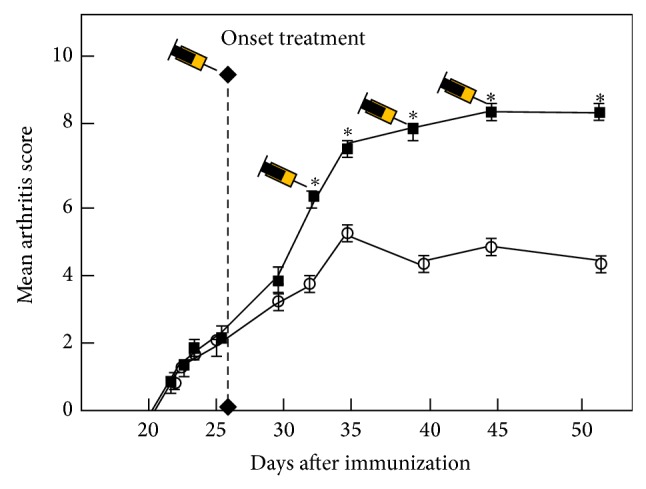
TNF*α*-blocker administration attenuates clinical CIA. Clinical scores in mice with CIA intraperitoneally treated from day 25 after immunization with PBS (control, C-group, closed squares) or TNF*α*-blocker (2 mg/week, E-group, open circles) once per week for four weeks. Values are the mean ± SD of 20 mice per group. Differences were significant at *P* < 0.001 (asterisks) for E-group versus C-group at indicated time points.

**Figure 2:**
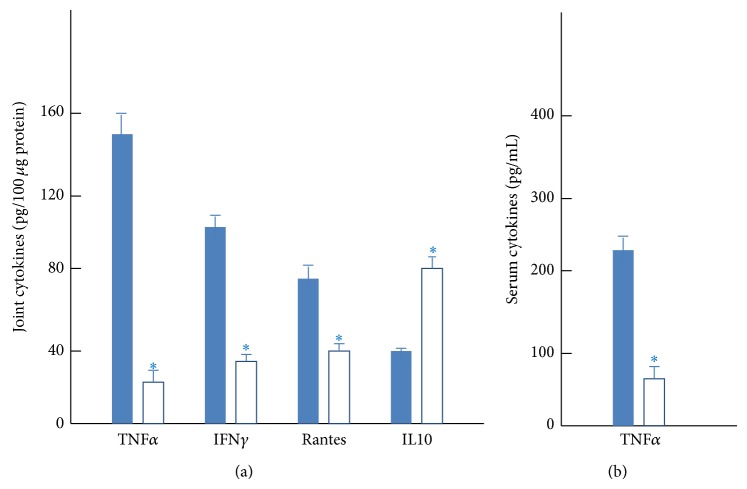
TNF*α*-blocker administration decreases inflammatory response in CIA. DBA1/J mice with established CIA (day 25 after immunization) were intraperitoneally injected with PBS (closed columns) or anti-TNF*α* (2 mg/week, open columns) once per week. Systemic and local expression of inflammatory mediators was assayed in protein extracts from joints of hind limbs (a) and sera (b) isolated at day 40 after immunization; *n* = 3 to 4 mice/group. ^∗^ *P* < 0.01 versus controls.

The clinical score was significantly attenuated in mice with CIA after 1 week of systemic treatment with the TNF*α*-blocker etanercept (*P* < 0.001) in comparison to untreated mice (Figure 1). This treatment also significantly reduced serum and joint tissue levels of Th1-mediated proinflammatory cytokines and increased levels of the anti-inflammatory cytokine IL10 (Figure 2).

**Figure 3:**
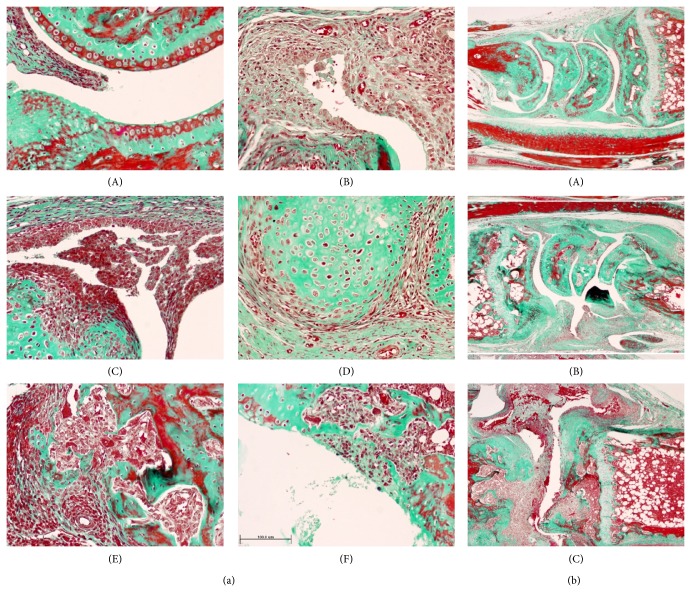
Morphological features of articular lesion in CIA model in DBA1/J mice. (a): (A) Unaffected joint. (B) Pannus. (C) Synovial hyperplasia with chronic inflammatory infiltrate. (D) Hyaline cartilage surrounded by spindle-shaped mesenchymal cells. (E) Bone destruction mediated by osteoclast activation. (F) Partial destruction of articular cartilage. Bar 100 *μ*m (Masson's trichrome, original magnification ×20). (b): (A) Limb without joint lesions. (B) Partial response to etanercept with persistence of pannus (E-group). (C) Intense joint lesion with pannus and chronic inflammatory infiltrate in articular cavity and partial destruction of bone tissue (C-group) (Masson's trichrome, original magnification ×4).

The histopathological study demonstrated a CIA induction efficiency of 95% in DBA1/J mice, which showed pannus formation and chronic lymphocytic/monocytic inflammatory infiltrate with acute phenomena (neutrophil leukocytes) that involved the joint soft tissues (Figure 3). These mice also showed the presence of necrotic cells in the intra-articular space, secondary destruction of the joint cartilage, and increased destruction of bone tissue through osteoclastic activation, with sporadic involvement of the bone medulla and extension of inflammatory infiltrate into periarticular soft tissues (Figure 3). Injection of etanercept in CIA mice moderately decreased most of the histopathological signs of arthritis, especially after four weeks of treatment (day 53 after immunization, Table 1). No significant differences in morphologic variables were observed between untreated and etanercept-treated mice after three weeks of treatment (day 46 after immunization, Table 1); however, stratification of the histopathological results according to the absence or presence of lesions (0 = absence, 1 = presence) showed that the etanercept treatment attenuated the progression of clinical inflammation in the limbs (mean of 0.55 in the etanercept group versus 0.85 in control group after 21 days of treatment; *P* < 0.003, Student's *t*-test).

**Figure 4:**
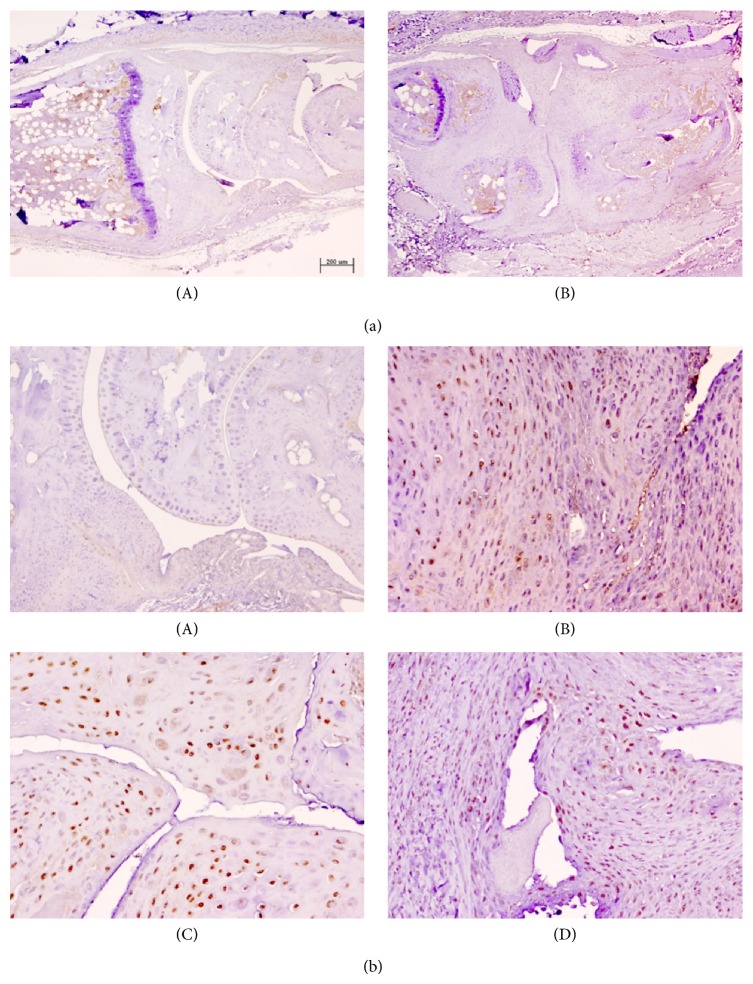
Immunohistochemical expression of Musashi-1 (Msi1) in CIA model in DBA1/J mice. (a): (A) Scant Msi1 expression was detected in chondrocytes, synoviocytes, and inflammatory cells in articular or periarticular lesions or in medullary bone tissue in joints with no morphological changes. (B) Nuclear expression of Msi1 in mesenchymal cells, articular capsule, and articular cartilage. Bar 200 *μ*m (micropolymer peroxidase-based method, original magnification ×4). (b): (A) Very scant nuclear Msi1 expression in joint with no morphological changes (E-group). (B), (C), and (D) Moderate Msi1 expression in mesenchymal cells, articular capsule, and articular cartilage in CIA-lesioned joints (C-group) (micropolymer peroxidase-based method, original magnification ×20).

The immunohistochemical expression of Msi1 was then investigated in different tissue components in the joints of untreated and etanercept-treated CIA mice. Msi1-specific staining was mainly observed at nuclear level in chondrocytes and spindle-shaped mesenchymal cells of the articular capsule and ligaments (Figure 4). Scant expression was detected in osteocytes, synoviocytes, or inflammatory cells in articular or periarticular lesions or in medullary bone tissue (Figure 4). No Msi1 expression was observed in striated muscle cells or mature adipocytes (Figure 4).

**Figure 5:**
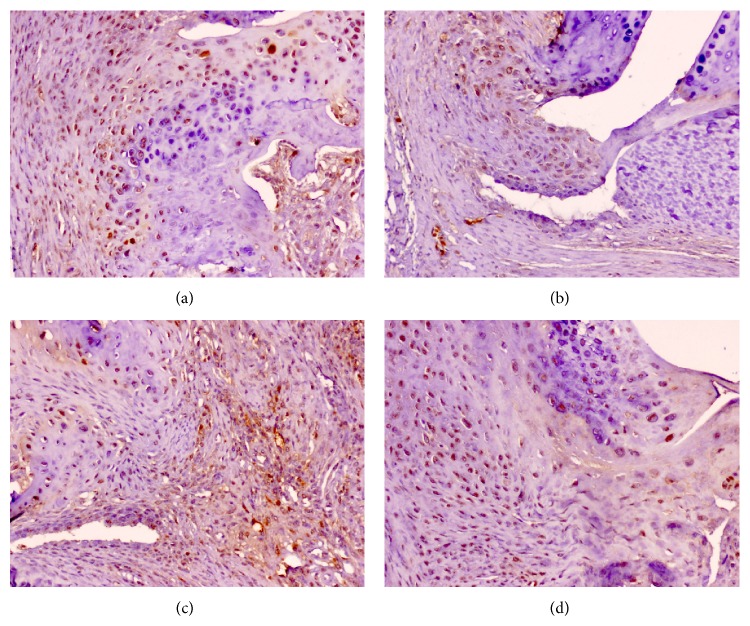
Representative immunohistochemical expression of Msi1 in E-group versus C-group in CIA model (DBA1/J mice). (a) Moderate Msi1 expression in mesenchymal cells, articular capsule, and articular cartilage in CIA-lesioned joints at 21 days after treatment (E-group); (b) moderate Msi1 expression in CIA-lesioned joints at 21 days after treatment (C-group); (c) joint Msi1 expression at 28 days after treatment (E-group); (d) joint Msi1 expression at 28 days after treatment (C-group) (micropolymer peroxidase-based method, original magnification ×20).

Quantitative analysis of Msi1 expression in the different groups revealed significantly higher expression in joints with CIA-induced articular lesions than in joints without lesions (*P* < 0.0001, Student's *t*-test, Figure 4). Significant positive correlations were also observed between the presence of osteoarticular lesion and Msi1 expression (Spearman's rho: 0.775, *P* = 0.001) and among Msi1 expression levels at the different sites (capsule, ligament, synovium, and cartilage) (Table 2). However, although a slight decrease in Msi1 expression was observed in affected joints in etanercept-treated CIA mice, no statistically significant differences in articular Msi1 expression were found between PBS-treated and etanercept-treated CIA mice after three (*P* = 0.449, Student's *t*-test) or four (*P* = 0.080, Student's *t*-test) weeks of treatment (Figure 5).

## 4. Discussion

This study confirmed that TNF*α*-blocker treatment attenuates CIA-induced histopathological lesions in the joints of CIA-susceptible DBA1/J mice and revealed, for the first time, a more intense expression of Msi1 protein in cartilage, ligament, articular capsule, mesenchymal cells, and osteocytes in CIA-lesioned versus nonlesioned joints.

Although no animal model of RA completely replicates the human disease, the CIA model employed in this study has been widely used for the testing and development of RA therapies [26–29]. In fact, the CIA induction rate was very high (>95%) in the present study. Likewise, DBA1/J mice were selected rather than other animals (e.g., rats) [30] because better outcomes are obtained in genetically modified strains and the arthritis is more similar to human RA [31, 32]. Major insights into the molecular mechanisms of inflammatory arthritis recently emerged from the study of murine models of RA-like disease using genetically deficient or transgenic mice or a combined murine model (K/BxA^g7^) that spontaneously develops both RA-like disease and atherosclerosis [33]. However, these studies may be limited by the differences between human and murine immune systems. Current efforts to develop an animal model that utilizes human immune cells will allow study of their function in the initiation and propagation of inflammatory arthritis [34].

RA is a chronic debilitating disease in which the induction of autoimmunity to collagen-rich joint components underlies the onset of the disease and the subsequent destructive inflammatory process. Progression of the autoimmune response implies the development of autoreactive Th1 (producing IFN*γ* and TNF*α*) and Th17 (producing Th17) cells, their entry into articular tissue, and the release of proinflammatory cytokines and chemokines, which promote macrophage and neutrophil infiltration and activation [3, 35, 36]. Excessive production by infiltrating inflammatory cells of inflammatory cytokines, free radicals, and extracellular matrix–degrading enzymes plays a critical role in cartilage damage and bone erosion. A desirable therapeutic approach would be to prevent the activation of inflammatory and autoimmune components. Our group previously demonstrated that 5-aminoisoquinolinone, a poly(ADP-ribose) polymerase-1 inhibitor, significantly reduces the incidence and severity of established CIA, completely abrogating joint swelling and cartilage/bone destruction by downregulating inflammation and the Th1 response [37]. The administration of TNF*α*-blocker to arthritic mice decreases the CII-specific Th1-mediated cytokine response through direct action on the synovium [38–40]. The present results confirm that treatment of established CIA with etanercept reduces articular levels of Th1 cytokines (IFN*γ* and TNF*α*) and inflammatory chemokines. This effect may be directly related to a decrease in inflammatory infiltrates in the joints of etanercept-treated mice. However, the fact that levels of the anti-inflammatory cytokine IL10 were increased by the injection of etanercept supports the proposition that this TNF*α*-blocker also promotes a bias towards a regulatory/anti-inflammatory response.

Etanercept and other blockers of TNF*α* action (infliximab, adalimumab, golimumab, and certolizumab pegol) offer specific anti-cytokine therapies but induce a general immunosuppressive action. Etanercept has become the drug of choice for late stage RA with reasonable safety, and the present study confirms that it attenuates the progression of histopathological lesions, as demonstrated in CIA murine models [12–17] and in the clinical setting [41, 42]. However, etanercept must be taken frequently, it is expensive, and it increases the susceptibility of the patient to infections [43].

The present study provides the first report on the osteoarticular expression of Msi1. No direct evidence has been published to date linking Msi1 with osteoarticular regeneration. Msi1 is involved in the regulation of self-renewal of stem cells. In order to maintain their unlimited capacity to divide, stem cells require controlled temporal and spatial protein expression. The Musashi family of RNA-binding proteins exerts this essential translational control (via repression and activation) in order to regulate multiple stem cell populations [44], and Msi1-dependent posttranscriptional enhancement of m-Numb is crucial in epithelial regeneration [45]. Nuclear expression of Msi1 was more intense in the presence of osteoarticular lesion and was not observed in nonlesioned joints, with or without anti-TNF*α* treatment. In previous studies on fractures in rats, we observed a marked increase in Msi1 expression in the reparative fibrocartilaginous tissue of the fracture callus (data not shown). Msi1 protein regulates the transcription and differentiation of mesenchymal stem cells; therefore, its presence in these tissues suggests its involvement in tissue repair and regeneration processes.

Our novel findings on the expression of Msi1 in osteoarticular tissues may support the participation of this protein in tissue regeneration processes and suggest the involvement of adult mesenchymal stem cells in the repair of these tissues. These data may serve as a basis for future investigations on repair processes in cartilaginous and bone tissues.

## 5. Conclusions

Treatment with etanercept attenuates the osteoarticular lesions in the murine model of CIA. The osteoarticular expression of Msi1 protein is increased in joints with CIA-induced lesion and absent in nonlesioned joints, suggesting that this protein is expressed when the lesions are produced in order to favor tissue repair.

## Figures and Tables

**Table 1:** Comparative study of morphological variables in DBA1/J mice after 21 and 28 days of starting treatment.

Variables	Control group	Etanercept group^*^	*P* values^†^	Control group	Etanercept group^*^	*P* values^†^
	Day 46 after immunization			Day 53 after immunization		
Number of joints	2.1 ± 0.69	1.8 ± 0.88	0.412	2.45 ± 1.01	1.9 ± 0.56	0.006
Pannus	1 ± 0.21	0.9 ± 0.35	0.449	0.7 ± 0.31	1 ± 0	0.467
B & C injury	1.8 ± 0.71	1.3 ± 0.78	0.278	2 ± 0.94	1.55 ± 0.68	0.045
Inflammation	1.5 ± 1	1.3 ± 1	0.661	1.55 ± 0.83	1.1 ± 0.69	0.036
Bone marrow	0.6 ± 0.47	0.5 ± 0.39	0.613	0.55 ± 0.42	0.55 ± 0.43	0.930
Soft tissue	0.15 ± 0.15	0.2 ± 0.21	0.557	0.35 ± 0.31	0.15 ± 0.15	0.010

Mean score	6.85 ± 2.95	5.8 ± 3.37	0.469	8 ± 3.55	6 ± 2.12	0.011

Values are expressed as mean ± standard deviation; ^*^treatment: etanercept 2 mg/week; B & C: bone and cartilage; ^†^Student's *t*-test. See Figure 3 for details.

**Table 2:** Spearman's correlation coefficient (rho) for Msi1 immunohistochemical expression between different articular components.

	Cartilage	Capsule	Ligament	Synovium
Cartilage	1	0.225^*^	0.308^**^	0.556^**^
Capsule		1	0.663^**^	0.389^**^
Ligament			1	0.260^*^
Synovium				1

^∗^Significant correlation at 0.05 (bilateral); ^**^significant correlation at 0.01 (bilateral).

## Abbreviations

AIQ: 5-Aminoisoquinolinone
CIA: Collagen-induced arthritis
CII: Type II collagen
IFN*γ*: Gamma interferon
Msi1: Musashi-1
RA: Rheumatoid arthritis
TNF*α*: Tumor necrosis factor alpha.
